# Supplementary material for: Patterns of Intron Gain and Loss in Fungi
Source: PLoS Biol. 2004 Nov 30;2(12):e422. doi: 10.1371/journal.pbio.0020422 (PMC532390; doi:10.1371/journal.pbio.0020422)
Supplement: Table S1 — Also available at http://genes.mit.edu/NielsenEtAl/. (4.3 MB ZIP). [file pbio.0020422.st001.zip › NielsenEtAl/html/1321.html]

AN4977.1.NCU04768.1.MG08880.1.FG08425.1


```
 CLUSTAL W (1.82) Multiple Sequence Alignments - Introns Inserted


Sequence 1: MG08880.1	602 aa
Sequence 2: FG08425.1	602 aa
Sequence 3: NCU04768.1	645 aa
Sequence 4: AN4977.1	633 aa
Alignment Length: 647 aa
Number Identitical Residues: 357 aa
Alignment Score (without introns) 16848


MG08880.1 	MSVSVKGAGRVCARI-------PSCRYSAPRLRTAALSSTSTAHYTTRPQRRLPRTTAAS
NCU04768.1	--MSTSARITSCLYRRAGSAGTTTSVQATRGLRTLSTPTINNISPASRSTRRPNVARQSY
FG08425.1 	--MPAPRPVSRCVQR-------SSQVLSRTQLR-PARSTFSPATIAARSSLTR-RTSTAL
AN4977.1  	--MASRGVVLRLLRR-------ETRPSPSRLSRPSSSLCISSRGSIATSGRRASVRCSAH
          	  :.                   :   .    *. :    .     : .         : 

MG08880.1 	WTQTRLFSVSRPRRNAS------EHESFDPRTVERESDEVDVCIVGGG1PAGLSAAIRLK
NCU04768.1	NPLLRTRQAAALSTTTQRRFADVADDAFDPNSIERESDEVDVCIVGGG~PAGLAAAIRLK
FG08425.1 	WSAAKVPMAARAFSTTSR--LRDDDTDFDPATVERESDEVDVCIVGGG~PAGLSAAIRLK
AN4977.1  	SRPIQVRAFSQSISRNLT----DENGHFDPRQVERESDEVDVCIVGGG1PAGLSAAIRLK
          	    :    :              .  ***  :*************** ****:******

MG08880.1 	QLANEAGNEDFRVLLLEKAGELGAHIVSGAVIQPTAIDELIPDWLSEDNPDRFEHATPAG
NCU04768.1	QLANEAGNDEFRVLLLEKAGEIGAHTLSGAVIEPSGIKELIPDWLDEENPNRFTGATPAG
FG08425.1 	QLANDAGNEDFRVLVLEKAGDLGAHILSGAVIQPTAINELFPDWLDENNPNRFEHATPAG
AN4977.1  	QLANEAGNEEFRVIVLEKAGEIGAHVLSGNVLEPSALEELFPDWLSEDNPSRFEGATPAK
          	****:***::***::*****::*** :** *::*:.:.**:****.*:**.**  **** 

MG08880.1 	TDRMRFLTKTKAIPLPAPPQMNNHGNYIVSLGQFTAWLGTRAEELGVEVYPGFAASEVIY
NCU04768.1	GDTMRFLTEKRAFWVPTPPQMHNKGNYIVSLSEFTKWLGERAEEVGVEVYPGFAASEVLY
FG08425.1 	TDRMRFLTKNSAIPIPAPPQMTNHGNYIVSLNQFVKWLGERAEEIGVEVYPGFAASEVVY
AN4977.1  	EDKMRFLTKNSSIPIPAPPQMNNHGNYIISLNELTKWLGERAEELGVEVYAGFAASEIVY
          	 * *****:. :: :*:**** *:****:**.::. *** ****:*****.******::*

MG08880.1 	KSDGSVLGVATNDLGIGRDGKPKDSFERGMEFHARCTLFAEGCHGSLTKQVTKKFDLRRD
NCU04768.1	KSDGSVKGVATNDLGLGRNGKPTDRFERGMEFHARVTLFGEGCHGSLSKQVINKYDLRRD
FG08425.1 	HADGSVKGVATNDLGVGRDGKPKETFERGMEFHARVTMFGEGCHGSLSKQVINKFDLRRD
AN4977.1  	NSDGSVKGVATNDLGVARDGQAKDTFERGMEFHARVTLLGEGCHGSLTKQVTKRYDLRRD
          	::**** ********:.*:*:..: ********** *::.*******:*** :::*****

MG08880.1 	SQPQTYGLGLKEVWEVDPAKFRKGEITHSLGYPLPKDVYGGAWMYHFGDNLVSLGLVVAL
NCU04768.1	SQPQTYGLGVKEVWEIQPEKFKKGEIFHSMGYPLPANTYGGAWMYHFGDNLVSIGLVTAL
FG08425.1 	SQHQTYGLGVKEVWEIDPAKFEKGLVVHSMGYPLPKDVYGGSFMYHFGENLVQIGLVTSL
AN4977.1  	SQPQTYGIGIKEVWEIQPEKFKSGEITHSMGYPLPKDTYGGAWMYHFGENMVSIGLVVGL
          	** ****:*:*****::* **..* : **:***** :.***::*****:*:*.:***..*

MG08880.1 	DYPNPWMSPYQEFQKMKKHPLYREVLEGGKCISYGARALIEGGFQSIPKVAFPGGALIGD
NCU04768.1	DYPNPWLSPYGEFQKMKQHPFYRHYLEGGKCISYGARALIEGGFQSIPKVAFPGGALIGD
FG08425.1 	DYTNPWMSPYQEFQKLKRHPLFRDVLEGGKCISYGARALIEGGFQSIPKVAFPGGALIGD
AN4977.1  	DYPNPWLSPYGEFQKLKHHPLYREVLEGGKCISYGARALNEGGYQSIPKCAFPGGALIGD
          	**.***:*** ****:*:**::*. ************** ***:***** **********

MG08880.1 	SAGFVNVPKVKGTHNAMKSGMLAAEAAWKALDTSENEGSVFLYDYESSLRQSSIWKELKE
NCU04768.1	SAGFVNVPKVKGTHNAMKSGMLAANAAWDALQDTNDSGSVFLYSYEDSLRNSSIWKELKE
FG08425.1 	SAGFVNVPKVKGTHNAMKSGMLAAEAAWTALSEKTDEGTVFLYDYENKLRDSSIWKELHE
AN4977.1  	SAGFLNVPKIKGTHTAMRSAILAAESTFAALRNDAG-GTVFLFDYEDSLRKSSIWKELYE
          	****:****:****.**:*.:***:::: **    . *:***:.**..**.******* *

MG08880.1 	VRNMRPSFHNPLGLYGGIMYSGLEAYVLKGRVPWTLKHKTTDHGATKPADQCEKIEYEKP
NCU04768.1	VRNMRPSFHSPLGLYGGIMYSGIDAFLLKGRVPWTFKHKVRDHEATKPADECEKIEYPKP
FG08425.1 	VRNMRPSFHTPLGLYGGIMYSGLEAYVFKGRVPWTLKHKTPDHASTLHADKVPKIEYEKP
AN4977.1  	VRNMRPSFSTPLGIYGGILYSGLEAYIFRGHTPWTLKHHGTDAAATKLASECEKIEYPKP
          	******** .***:****:***::*::::*:.***:**:  *  :*  *.:  **** **

MG08880.1 	DGKITFDILTSVSRTGTNHEEDQPVHLQVKDWDRHALETWPKFKGLENRFCPAGVYEYVE
NCU04768.1	DGKITFDILTSVSRTGTNHEEDQPVHLQVEDWDQHTKETYPKYKGVENRFCPAGVYEYVE
FG08425.1 	DGKLTFDILTSVSRTGTNHEEDQPVHLQVKDWDAHTESTYPPYKGMENRFCPAGVYEYVE
AN4977.1  	DGKISFDILTSVSRTGTNHEEDQPVHLQVEDWDKHTDIAWPKYKGVENRFCPAGVYEYVE
          	***::************************:*** *:  ::* :**:**************

MG08880.1 	DQSKELGVRFQINAQK~--------------------------------
NCU04768.1	DATKKEGVRFQINAQN2CIHCKTCDIKAPNQDINWQVPQGGEGPKYYMT
FG08425.1 	DESKPHGVRFQINAQK~--------------------------------
AN4977.1  	DSSKPHGVRFQINAQN2CIHCKTCDIKVPTQDINWQTPQGGEGPKYFKT
          	* :*  *********: .  ..:.. . .... . . ........   :
```
